# Fossils indicate marine dispersal in osteoglossid fishes, a classic example of continental vicariance

**DOI:** 10.1098/rspb.2024.1293

**Published:** 2024-08-14

**Authors:** Alessio Capobianco, Matt Friedman

**Affiliations:** ^1^ GeoBio-Center LMU, Ludwig-Maximilians-Universität München, Munich, Germany; ^2^ Department of Earth and Environmental Sciences, Palaeontology & Geobiology, Ludwig-Maximilians-Universität München, Munich, Germany; ^3^ Department of Earth and Environmental Sciences, University of Michigan, Ann Arbor, MI, USA; ^4^ Museum of Paleontology, University of Michigan, Ann Arbor, MI, USA

**Keywords:** biogeography, dispersal, bonytongue fishes, vicariance, FBD, total-evidence

## Abstract

The separation of closely related terrestrial or freshwater species by vast marine barriers represents a biogeographical riddle. Such cases can provide evidence for vicariance, a process whereby ancient geological events like continental rifting divided ancestral geographical ranges. With an evolutionary history extending tens of millions of years, freshwater ecology, and distribution encompassing widely separated southern landmasses, osteoglossid bonytongue fishes are a textbook case of vicariance attributed to Mesozoic fragmentation of the Gondwanan supercontinent. Largely overlooked fossils complicate the clean narrative invoked for extant species by recording occurrences on additional continents and in marine settings. Here, we present a new total-evidence phylogenetic hypothesis for bonytongue fishes combined with quantitative models of range evolution and show that the last common ancestor of extant osteoglossids was likely marine, and that the group colonized freshwater settings at least four times when both extant and extinct lineages are considered. The correspondence between extant osteoglossid relationships and patterns of continental fragmentation therefore represents a striking example of biogeographical pseudocongruence. Contrary to arguments against vicariance hypotheses that rely only on temporal or phylogenetic evidence, these results provide direct palaeontological support for enhanced dispersal ability early in the history of a group with widely separated distributions in the modern day.

## 1. Introduction

Why closely related terrestrial and freshwater organisms can be found in landmasses separated by vast stretches of sea is an outstanding question that traces back to the very beginnings of evolutionary biology as a discipline [1,2]. Proposed models to explain these disjunct geographical distributions fall into two broad categories: vicariance, whereby ancient geological events such as continental breakup created marine barriers and divided ancestral geographical ranges, and long-distance dispersal, whereby organisms dispersed over those barriers more recently. While vicariance became the dominant framework to interpret inter-continental distributions after the wide acceptance of plate tectonics [3], the last 20 years have seen a resurgence of long-distance dispersal as a plausible and even widespread biogeographical process [4,5]. However, a long-distance dispersal framework has been criticized in the past for relying on ad hoc explanations and negative evidence, and for not proposing testable hypotheses [6–8]. Even though most of these critiques have been countered thanks to methodological and statistical advances [5,9], the mechanisms by which terrestrial and freshwater taxa managed to cross oceanic barriers remain unclear, with positive evidence for transoceanic dispersals proving particularly elusive. Further complications emerge when considering the fossil record, as extinct species can often be found in geographical areas outside the distribution of their living relatives. Some notable cases include marsupials [10], lungfishes [11] and gars [12], all of which show more complex past geographical distributions. Because of such patterns, the importance of fossils for biogeographical studies has been appreciated for more than a century [2]. Nevertheless, inclusion of fossil data in model-based biogeographical analyses of extant taxa remains limited to just a few remarkable examples in the literature [12–21].

**Figure 1.**
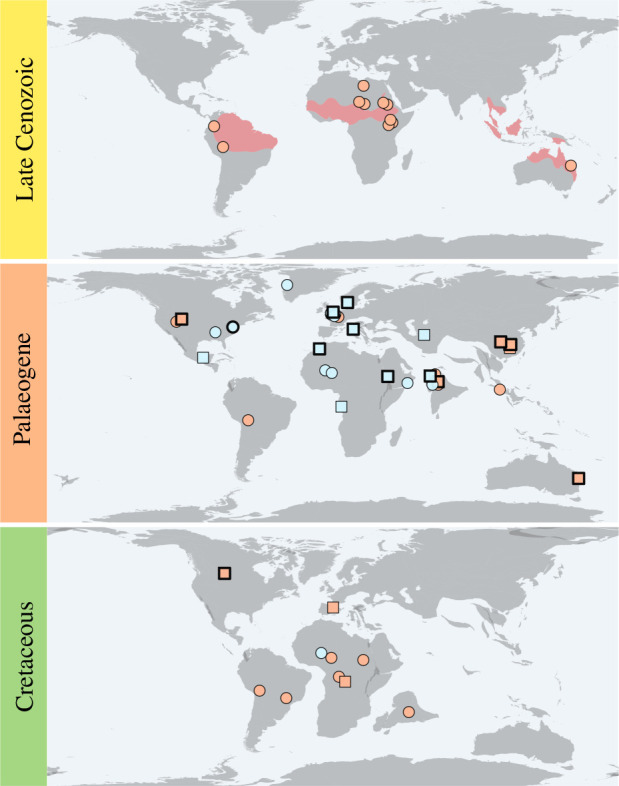
Geographical distribution of extinct and extant osteoglossid bonytongues. Fossil occurrences are divided by preservation state (*circles*: fragmentary and disarticulated fossils; *squares*: articulated fossils) and palaeoenvironment (*orange fill*: freshwater deposits; *light blue fill*: marine deposits). Fossil occurrences with thicker borders indicate where the extinct osteoglossids included in the phylogenetic and biogeographical analyses have been found. The red area in the late Cenozoic map displays the current geographical distribution of extant osteoglossids. Palaeogeographic maps at 0, 50 and 85 Ma were generated in the R package mapast under the MULLER2016 model [30]. Fossil osteoglossid occurrences from [26,31,32]. Geographical distribution of extant osteoglossids from [33].

Freshwater fishes in particular provide a model system in historical biogeography owing to how evolving geomorphological and tectonic features can present either hard barriers or favourable corridors to their dispersal (e.g. [22–24]). Inclusion of fossils in freshwater fish biogeography is limited by the geographical and temporal patchiness of freshwater deposits with the potential for exceptional preservation of relatively small, delicate vertebrates. Osteoglossomorphs or bonytongue fishes (Osteoglossomorpha) are a celebrated example of a freshwater fish clade with an unusually good fossil record, encompassing every continent except Antarctica and extending to the Late Jurassic–Early Cretaceous (*ca* 160−100 Ma) [25,26]. Owing to the wide distribution and exclusively freshwater ecology of extant osteoglossomorphs, they have been often viewed as a textbook example of vicariance, at the level either of the entire clade or of some of their subclades, including Osteoglossidae [7,27–29]. Osteoglossidae is today represented by four genera living in South America, Africa, Southeast Asia and Australia. These fishes, commonly called arapaimas and arowanas, include some of the largest freshwater fishes in the world and are popular staples of public aquariums for their charismatic and ‘prehistoric’ appearance. Remarkably, fossils of osteoglossids are found not only outside their current geographical range but also outside their present environmental tolerance, with several extinct species known from marine deposits dating to the early Cenozoic (66−40 Ma) (figure 1) [25,26,31,32]. Thus, marine long-distance dispersal represents a possible explanation for the modern disjunct distribution of osteoglossid bonytongues [25,26,31,32,34–38], but this hypothesis has never been tested within a phylogenetic framework under a biogeographical model.

Here, we estimate ancestral geographical ranges and ancestral habitats for bonytongue fishes under a new total-evidence phylogenetic hypothesis including all extant genera and 32 extinct species of bonytongues. We aim to answer three key questions about the evolutionary history of bonytongue fishes: (i) what are the phylogenetic relationships of extinct marine bonytongues; (ii) what are the major patterns of historical biogeography within the clade, and are they consistent with a vicariance or long-distance dispersal framework; and (iii) are extant freshwater osteoglossids (arapaimas and arowanas) descended from marine ancestors? By doing so, we provide an unprecedented example of how fossil data can dramatically revise biogeographical scenarios that would be strongly supported by the examination of extant species only.

## 2. Methods

### (a) Morphological dataset

The morphological matrix used for the total-evidence phylogenetic analysis of this study is a modification of the morphological dataset of Capobianco *et al*. [32], with the novel addition of 2 extant and 14 extinct species. The list of newly added taxa, complete with the list of specimens and literature used to determine the scoring of morphological characters, is available in the electronic supplementary material. To make the morphological matrix compatible with the molecular dataset for a total-evidence analysis, the taxonomic resolution of extant operational taxonomic units (OTUs) was changed from genus level to species level. In the cases where an extant genus was represented by multiple species in the molecular dataset, we assigned the morphological character scoring for that genus to the species that was examined by the original scorer of those characters (e.g. [39]) and/or to the species that we could examine through osteological specimens or micro-computed tomography (µCT) data. Thus, we changed OTUs from the matrix in [32] as follows: *Campylomormyrus* → *C. tamandua*; *Chitala* → *C. chitala*; *Hiodon* → *H. alosoides* and *H. tergisus*; *Osteoglossum* → *O. bicirrhosum*; *Papyrocranus* → *P. afer*; *Petrocephalus* → *P. simus*; *Scleropages* → *S. formosus*, *S. leichardti* and *S. jardinii*. Notably, the morphological characters of this matrix are mostly invariant for congeneric extant species (with the exception of *Scleropages*; see electronic supplementary material), because they were defined to capture morphological variation across Osteoglossomorpha with the purpose of resolving relationships between major bonytongue clades [32,39,40]. The morphological matrix, which ultimately comprised 96 characters for 53 OTUs (33 extinct and 20 extant), was assembled and edited in Mesquite v. 3.61 [41].

### (b) Molecular dataset

The molecular data matrix was assembled by integrating part of the genomic dataset of [38] with semi-automated extraction of DNA sequences from Genbank (via the NCBI platform) and BOLD (Barcode Of Life Data system), using functions from the R package regPhylo [42]. A total of 12 DNA markers were selected: 2 protein-coding mitochondrial markers (*coI, cytb*), 2 non-protein-coding mitochondrial markers (12S rRNA, 16S rRNA) and 8 protein-coding nuclear markers (*rag1*, *rag2*, *glyt*, *ficd*, *megf8*, *pdzd8*, *suox*, *vcpip1*). Details of the assembly of the molecular dataset are available in the electronic supplementary material. As the phylogeny and biogeography of the hyperdiverse Mormyridae (elephantfishes) are not the main focus of this study, we subsampled mormyrids to maximize phylogenetic coverage of the clade while reducing computational burden. The final molecular dataset comprised 14 084 nucleotides for 63 OTUs—including all extant osteoglossomorph genera and 23.4% of all extant osteoglossomorph species—with 87% matrix completeness at the marker level.

### (c) Total-evidence phylogenetic analysis

We combined the morphological and molecular matrices to generate a total-evidence dataset including 96 OTUs (33 extinct and 63 extant). A partitioning scheme for the molecular portion of the dataset was determined using PartitionFinder 2 [43], with greedy search algorithm and allowing partitions based on codon position in the 10 protein-coding markers. As a result, the best partitioning scheme included eight molecular partitions (electronic supplementary material). The morphological portion of the dataset was treated as a separate additional partition.

An unrooted, non-time-calibrated tree was first estimated in the software MrBayes [44] to provide a starting tree for the time-calibrated phylogenetic analysis (electronic supplementary material, figure S1). *Amia calva* was constrained as the outgroup to all other OTUs, while the other three outgroups (*Elops saurus*, *Dorosoma cepedianum* and †Ellimmichthyiformes) were constrained to be outside of total-group Osteoglossomorpha, which included the remaining 93 OTUs. A GTR + **Γ **+ I substitution model was applied to each molecular partition, while an Mk*v* substitution model was applied to the morphological partition. The Metropolis-coupled Markov chain Monte Carlo (MCMCMC) was set up as 2 runs with 4 chains each, running for 50 million generations and sampling every 10 000. Parameter summaries and the ‘Allcompat’ summary consensus tree were calculated using a 50% burn-in fraction.

After the unrooted analysis, we ran a Bayesian time-calibrated phylogenetic analysis in MrBayes under a skyline fossilized birth–death (SFBD) model [45]. The analysis was run on the PalMuc high-performance computing (HPC) cluster at LMU Munich. As in the unrooted analysis, a GTR + **Γ **+ I substitution model was applied to each molecular partition, and an Mk*v* substitution model was applied to the morphological partition, with all characters unordered. A relaxed clock model with independent gamma rates (IGR) was applied separately to the mitochondrial, nuclear and morphological portions of the dataset, to allow for rate variation across branches. The SFBD tree model was set up to allow fossil sampling rate to vary between four time intervals: pre-Cretaceous (up to 145 Ma), Cretaceous (145−66 Ma), Palaeocene–Eocene (66−33.9 Ma) and Oligocene–Recent (33.9−0 Ma). We did not allow extinct taxa to be recovered as sampled ancestors, as incorrectly recovered sampled ancestors (false positives) might heavily bias downstream ancestral state reconstructions like the biogeographical analyses performed in this study, owing to their zero-length branches. More detailed information about the settings of our time-calibrated analysis, including choice of clock rate and tree age priors, and internal node constraints, can be found in the electronic supplementary material. Tip ages of extinct taxa were assigned a prior uniform distribution ranging from minimum to maximum possible age of the fossil deposit where that taxon has been found. A list of all fossil tip ages with references can be found in the electronic supplementary material. The MCMCMC was set up as 2 runs with 4 chains each, running for 450 million generations and sampling every 1000, with 10% burn-in fraction. Convergence of parameters between the two runs was checked in Tracer [46] by comparing their posterior estimates and by calculating their effective sample sizes, which were >200 for all parameters. Posterior tree files were resampled as one tree every 10 000 generations before calculating the ‘Allcompat’ summary consensus tree (a majority rule tree showing all compatible taxon bipartitions). The phylogenetic position of extinct taxa with respect to extant ones across the posterior distribution of trees was evaluated using the function ‘create.rogue.plot’ from the R script RoguePlots [47].

### (d) Biogeographical analysis

The biogeographical analysis was set up and run in the R package BioGeoBEARS [48]. Continental land masses were divided into seven areas encompassing the whole distribution of extant and extinct Osteoglossomorpha and corresponding to major biogeographical regions for extant freshwater fishes [22]: Nearctic, Neotropical, Ethiopian, Palaearctic, Sinean, Indo-Malayan (or Oriental) and Australian. For simplicity, we refer to these regions, respectively, as North America, South America, Africa, Europe, continental Asia, Indo-Malaya and Australia in the main text and figures. Additionally, we considered the marine realm as an additional geographical area (bringing the total to eight areas), and extinct taxa found in marine deposits were scored as occurring exclusively in the marine area.

We also tested an alternative scoring scheme where extinct taxa found in marine deposits were scored as belonging to the continental biogeographical regions where their fossils have been found (electronic supplementary material). This alternative scoring scheme, while providing more granular information about the geographical distribution of marine bonytongues, has the disadvantage of confounding marine and freshwater biogeographical regions (e.g. a marine taxon found in North America could come from the eastern Pacific or the western Atlantic, two very distinct regions with different biogeographical affinities). Thus, we will primarily refer to results obtained under the first scoring scheme.

The maximum number of areas that could be occupied by a single lineage at any one point was fixed to 3, to reduce the number of allowed geographical states and reduce computational time. We further restricted the state space of the analysis by removing all geographical states corresponding to the marine realm plus two continental regions, but we kept states corresponding to the marine realm plus one continental region to potentially allow for a euryhaline (freshwater + marine) condition.

We applied the standard biogeographical models implemented in BioGeoBEARS on the ‘AllCompat’ summary consensus tree obtained by the total-evidence phylogenetic analysis. These models include DEC (Dispersal–Extinction–Cladogenesis [49]), DIVALIKE (a likelihood interpretation of the parsimony DIVA, DIspersal Vicariance Analysis model [50]) and BAYAREALIKE (a simplified likelihood interpretation of the Bayesian model implemented in the software ‘BayArea’ [51]). Additionally, we ran variants of these three models that include a jump dispersal parameter, *j*, which allows for founder-event jump dispersal during cladogenesis (see [52] for a critique of the DEC + model, and [53] for a partial response to that critique). Standard tools for statistical model comparison [54]—including likelihood ratio test for pairs of nested models and Akaike information criterion for small sample sizes (AICc)—were used to evaluate model support.

To test how phylogenetic uncertainty impacts the results of the biogeographical analysis, we applied the best-fitting biogeographic model to 200 phylogenies sampled from the Bayesian posterior distribution. The results from these 200 analyses were summarized by recording marginal ancestral area reconstructions for eight clades: total-group Osteoglossomorpha (root node), crown Osteoglossomorpha (*Hiodon alosoides + Osteoglossum bicirrhosum* node), crown Osteoglossiformes (*Pantodon buccholzi + Osteoglossum bicirrhosum* node), crown Osteoglossidae (*Osteoglossum bicirrhosum + Arapaima gigas* node), crown Osteoglossinae (*Osteoglossum bicirrhosum + Scleropages formosus* node), crown Arapaiminae (*Arapaima gigas + Heterotis niloticus* node), crown Notopteroidei (*Notopterus notopterus + Mormyrus ovis* node) and crown Notopteridae (*Notopterus notopterus + Papyrocranus afer* node). For these clades, we calculated average marginal probabilities for each possible state, corresponding to empirical Bayesian posterior probabilities.

To explore how the inclusion of fossil data impacts biogeographical inference, we ran the standard BioGeoBEARS models listed above on the Bayesian consensus tree pruned of all extinct taxa. We compared marginal ancestral states found in this extant-only analysis with marginal ancestral states recovered from the previous integration of 200 phylogenies with extinct taxa from the posterior distribution.

To examine dispersal directionality between the eight biogeographical regions, we performed biogeographical stochastic mapping (BSM) [55] as implemented in BioGeoBEARS. We simulated 100 BSMs under the best-fitting parameters with the DEC + *j* model for each of the 200 phylogenies previously sampled from the Bayesian posterior distribution. The average number of dispersal events from and to each biogeographical region, comprehensive of both anagenetic and cladogenetic (jump) dispersal, were tabulated for each of the 200 posterior phylogenies and then averaged across phylogenetic uncertainty by calculating their mean.

### (e) Ancestral habitat estimation

As a complementary approach to reconstruct transitions between freshwater regions and the marine realm, we applied ancestral state estimation (ASE) on a binary ecological character (freshwater versus marine) in the R package corHMM [56]. Extinct taxa were assigned to the freshwater or marine state depending on the palaeoenvironmental reconstruction of the fossil deposits they have been found in (electronic supplementary material). We estimated marginal ancestral states under an all-rates-different (ARD) model on the Bayesian consensus tree, with root state probabilities based on the estimated transition rates (root.p=“yang” flag in the corHMM function). To estimate the number of transition events from freshwater to marine environments and *vice versa*, we used the makeSimmap function to generate 10 000 stochastic character mappings on the Bayesian consensus tree using the maximum likelihood transition rate matrix previously calculated under the ARD model.

In order to account for phylogenetic uncertainty when estimating freshwater–marine transitions, stochastic character mapping was also performed on a random sample of 1000 phylogenies from the Bayesian posterior distribution in the R package phytools [57]. Ten stochastic character mappings were generated for each sampled phylogeny under the ARD model with estimated root state probabilities. The simulated numbers of transition events from freshwater to marine environments and *vice versa* were summarized by plotting histograms and by calculating mean and relevant quantiles.

## 3. Results

### (a) Phylogenetic relationships

**Figure 2.**
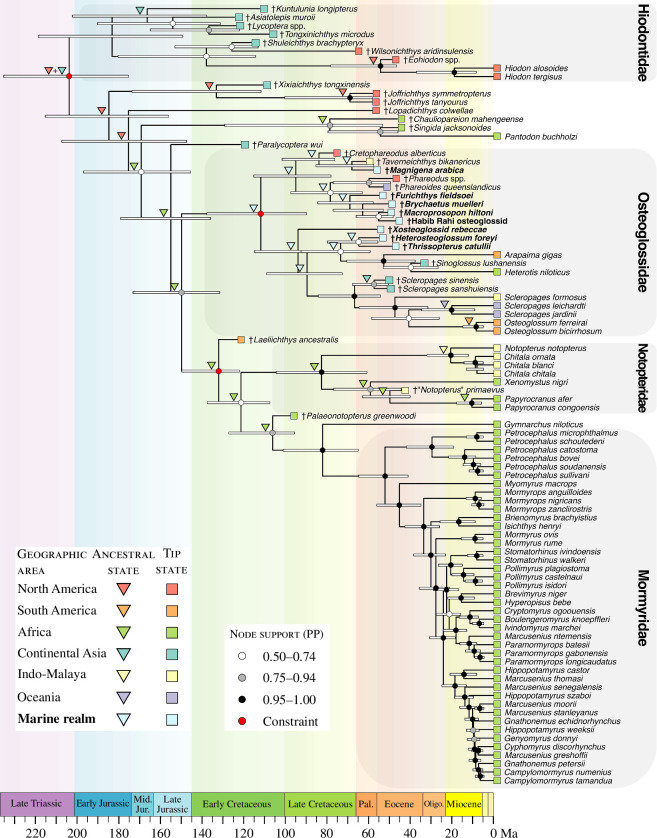
Time-calibrated phylogeny of Osteoglossomorpha. The phylogeny plotted here is the ‘AllCompat’ summary consensus tree from the Bayesian total-evidence analysis. Tips are coloured according to geographical distribution. Taxa found in marine settings are highlighted in bold. Coloured triangles at internal nodes represent the most likely ancestral geographical area under the DEC+*j* model when it is at least 3.2 times more likely than the second most likely geographical area, indicating substantial strength of evidence under a Bayes factor framework. Coloured triangles at internal nodes for which all descendant tips and the immediately ancestral node inhabit the same area were masked to avoid figure cluttering. Circles at internal nodes indicate node support as Bayesian posterior probabilities of clades when equal to or larger than 0.50. White bars represent 95% highest posterior densities (HPDs) of node ages. Boxes encompass terminals belonging to total group (crown group + stem group) of named clades.

Most major phylogenetic relationships recovered in the Bayesian total-evidence time-calibrated phylogenetic analysis (figure 2) are compatible with previous morphological and molecular studies (e.g. [38–40,58–61]; see [25] for a review of osteoglossomorph systematics). These include mooneyes (Hiodontidae) as living sister group to all other extant bonytongues (Osteoglossiformes); elephantfishes (Mormyridae) and the aba (Gymnarchidae) as closely related to Old World knifefishes (Notopteridae); and arapaimas and relatives (Arapaiminae) being closely related to arowanas (Osteoglossinae) and forming the clade Osteoglossidae. The African butterflyfish *Pantodon* was recovered as living sister group to all other extant Osteoglossiformes, a position that is not supported by morphological characters alone [32,39,40] but which is often found in molecular phylogenetic analyses (e.g., [38,61]). Intergeneric relationships within the species-rich Mormyridae match those recovered by Peterson *et al*. [38].

The posterior probabilities of several nodes forming the ‘backbone’ of the osteoglossomorph

tree are extremely low owing to the uncertain position of several extinct taxa (figure 2). However, even when posterior probabilities of nodes including extinct taxa are very low, those taxa might consistently resolve in few distinct positions compared with extant taxa [47]. Hence, exploring the position of extinct taxa with respect to extant ones across the posterior distribution of phylogenies can be more informative than just examining node supports on a consensus tree (electronic supplementary material, figure S2). Relationships of non-osteoglossid extinct taxa broadly match previous hypotheses, and are discussed in further detail in the electronic supplementary material.

All marine taxa included in the analysis (†*Brychaetus*, †*Furichthys*, †*Heterosteoglossum*, †*Macroprosopon*, †*Magnigena*, †*Thrissopterus*, †*Xosteoglossid* and the undescribed Habib Rahi taxon) are recovered as either crown or stem members of Osteoglossidae. Some of them are often grouped together with extinct freshwater taxa from various continents (†*Phareodus*, †*Phareoides*, †*Taverneichthys* and †*Cretophareodus*), making up the clade †Phareodontinae (*sensu* [32]). The position of †Phareodontinae within Osteoglossidae is not well resolved, although a stem osteoglossid position is more favoured than other placements. The marine taxa †*Heterosteoglossum* and †*Thrissopterus* are most often recovered as stem members of Arapaiminae. †*Sinoglossus* from the late Eocene–Oligocene (*ca* 38–23 Ma) of China is either reconstructed as sister to the African *Heterotis* or as sister to the South American *Arapaima*. The Eocene Chinese species of *Scleropages* (†*S. sinensis* and †*S. sanshuiensis*) resolve almost always as stem Osteoglossinae, suggesting they might represent members of an extinct genus distinct from *Scleropages*.

### (b) Evolutionary timescale

Our Bayesian time-calibrated phylogenetic analysis provides the most comprehensive assessment of the evolutionary timescale of bonytongue fishes to date. Osteoglossomorph origin is estimated to occur between the Late Triassic and the Early Jurassic (95% highest posterior density (HPD) = 235.3–175.3 Ma). This is older than previous fossil-based estimates [26], but slightly younger than estimates based only on molecular data [38,59,62]. Crown Osteoglossiformes appear to have originated in the Jurassic (95% HPD = 196.8–145.4 Ma), while the divergence between the two largest bonytongue clades (Osteoglossidae on one side, Mormyroidei and Notopteridae on the other) occurred between the Middle Jurassic and the very beginning of the Early Cretaceous (95% HPD = 173.1–131.4 Ma). Old World knifefishes (Notopteridae) diverged from elephantfishes and relatives (Mormyroidei) in the Early Cretaceous (95% HPD = 137.5–107.3 Ma). The divergence between extant African and Asian knifefishes likely happened in the Late Cretaceous (95% HPD = 104.3–60.6 Ma), significantly postdating the fragmentation of East and West Gondwana [37]. The hyper-diverse elephantfishes started diversifying between the Palaeocene and the early middle Eocene (95% HPD = 64.7–40.9 Ma), with most divergences between extant genera occurring in the Oligocene and Miocene.

The origin of osteoglossid bonytongues is estimated to occur between the Early Cretaceous and the early Late Cretaceous (95% HPD = 137.7–89.7 Ma). The divergence between Arapaiminae and Osteoglossinae (crown Osteoglossidae) likely happened in the Late Cretaceous before the Maastrichtian (95% HPD = 108.9–72.5 Ma). The split between the South American *Arapaima* and the African *Heterotis* likely occurred between the Maastrichtian and the middle Eocene (95% HPD = 70.4–37.5 Ma), while the split between the Southeast Asian *Scleropages formosus* and the South American *Osteoglossum* is estimated to occur in the Palaeocene–Eocene interval (95% HPD = 64.5–31.3 Ma). These divergences between osteoglossid genera inhabiting disjunct continents postdate the fragmentation of Gondwanan landmasses, except for the separation between South America, Antarctica and Australia, which likely occurred during the Palaeocene–Eocene interval [63].

### (c) Historical biogeography and freshwater–marine transitions

**Figure 3.**
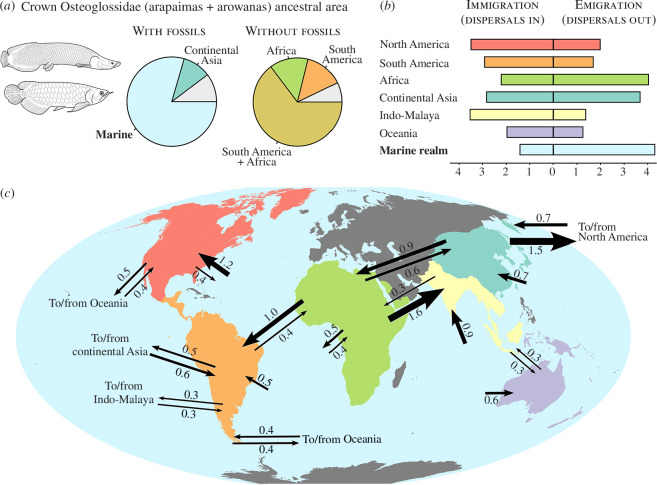
Historical biogeography of bonytongue fishes integrated over phylogenetic uncertainty. Marginal probabilities and numbers of dispersal events shown in this figure have been integrated over a random sample of 200 phylogenies from the Bayesian posterior distribution. (*a*) Marginal probabilities of ancestral biogeographical area under the DEC + *j* model for crown Osteoglossidae when fossils are included in (*left*) or excluded from the analysis (*right*). (*b*) Average number of dispersal events into (immigration) and from (emigration) biogeographical areas, calculated under biogeographical stochastic mapping (BSM) and integrated over phylogenetic uncertainty. (*c*) Directionality of dispersal between biogeographical areas, calculated under BSM. Arrow thickness is proportional to the average number of dispersal events, indicated by the number next to the arrow. Arrows from one area to another are not shown when the average number of dispersal events is lower than 0.3.

In a biogeographical analysis excluding fossils, we find a pattern broadly consistent with a continental vicariance scenario that matches previous hypotheses for the biogeographical history of bonytongues [7,27–29]. Major biogeographical findings include a West Gondwanan plus North American ancestral distribution for crown osteoglossomorphs, and a West Gondwanan ancestral distribution for crown osteoglossids (figure 3*a*; electronic supplementary material, figures S3 and S4). Both are associated with major vicariant splits: the split between Laurasia and Gondwana leads to the North American *Hiodon* on one side and to the Gondwanan Osteoglossiformes on the other, while the split between Africa and South America leads to *Heterotis* on one side and to South American osteoglossids on the other.

Fossils radically revise this picture of osteoglossomorph biogeography. Our two approaches to considering marine associations in extinct osteoglossomorphs yield consistent and complementary inferences about the group’s biogeographical and ecological history. When marine settings are treated as a biogeographical region, we find a marine ancestral distribution for crown osteoglossids under the DEC + *j* model (figure 2). This striking result is robust to phylogenetic uncertainty (figure 3*a*). The ancestral distributions of both crown Osteoglossinae and crown Arapaiminae are not reconstructed as marine and are instead uncertain between the freshwater geographical areas in which their members are found (Indo-Malaya, Australia and South America for crown Osteoglossinae; Africa, continental Asia and South America for crown Arapaiminae). However, this might represent a conservative result owing to the likely under-parameterized nature of the biogeographical models used here (see §4). Other key biogeographical findings include a Laurasian (Asia + North America) origin for crown osteoglossomorphs, followed by a dispersal from Laurasian landmasses to Africa leading to the origin of Osteoglossiformes. The specific source of this dispersal (North America or Asia) is sensitive to topological differences in the posterior distribution of phylogenies.

**Figure 4.**
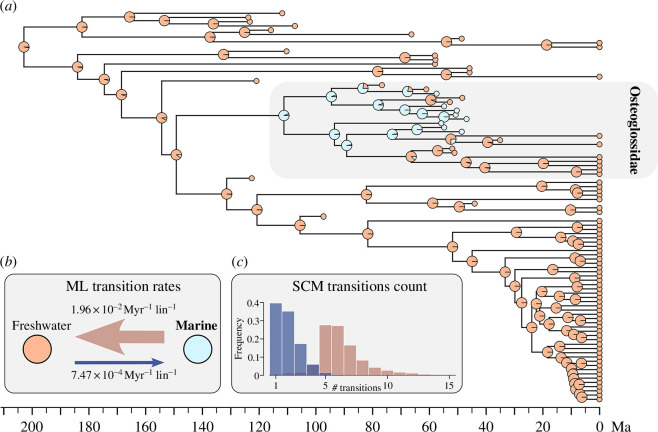
Ancestral habitat estimation for bonytongue fishes. (*a*) Marginal ancestral states under an all-rates-different (ARD) model on the Bayesian consensus tree of Osteoglossomorpha. Orange fill indicates freshwater environment, while light blue fill indicates marine environment. Tip identities are the same as in figure 2, with light grey box encompassing terminals belonging to total-group Osteoglossidae. (*b*) Maximum-likelihood (ML) estimates of transition rates between freshwater and marine environments, expressed per million years per lineage (Myr^−1^ lin^−1^). (*c*) Distribution of the inferred number of environmental transitions under stochastic character mapping (SCM) on the Bayesian consensus tree of Osteoglossomorpha. Freshwater-to-marine transitions are in dark blue and marine-to-freshwater transitions are in rosy brown.

When marine and freshwater settings are treated as a binary ecological character evolving under an ARD Markov model, we also find strong support for an ancestral marine ecology in crown osteoglossids (figure 4). Rates of transition between marine and freshwater environments are strongly asymmetric, with the marine-to-freshwater transition rate estimated to be more than one order of magnitude larger than the freshwater-to-marine rate (freshwater-to-marine rate = 7.47 × 10^−4^ Myr^−1^ per lineage; marine-to-freshwater rate = 1.96 × 10^−2^ Myr^−1^ per lineage). This asymmetry is reflected in the number of transitions calculated across 1000 stochastic character mappings simulated under maximum-likelihood parameter estimates on the Bayesian consensus tree (figure 4*c*). Bonytongue fishes invaded marine environments on average 1.9 times (mode = 1, corresponding to the marine invasion associated with the origin of the osteoglossid lineage), but they re-entered freshwater environments on average 6.3 times (mode = 5). Comparable results are found when considering phylogenetic uncertainty by simulating stochastic character mappings across a sample of the Bayesian posterior distribution of trees (electronic supplementary material, figure S5).

## 4. Discussion

### (a) Biogeographical history of Osteoglossomorpha

A comprehensive picture of the biogeographical history of Osteoglossomorpha can be reconstructed by integrating the divergence-time estimates and ancestral eco-geographical reconstructions obtained in this study with Earth’s geo-palaeontological history. The ancestral osteoglossomorph likely lived in freshwater environments in the Laurasian supercontinent between the Late Triassic and Early Jurassic. Several early-diverging lineages of osteoglossomorphs are found in bothAsian and North American fossil deposits, hinting at multiple dispersals between these continents during the Mesozoic. Faunal exchanges between Asia and North America in the Jurassic and Cretaceous are strongly supported for multiple groups of continental organisms, including dinosaurs, mammals, and several other freshwater fish taxa [26,64]. Crown Osteoglossiformes are here inferred to have an African origin in the Jurassic. Strikingly, while no articulated bonytongue fossil has ever been found in African Jurassic deposits, fragments of scales (squamules) similar to those of modern osteoglossiforms have been recovered from the Middle Jurassic Anoual Formation of Morocco [65], matching our age estimate for the origin of Osteoglossiformes and providing a potential earliest occurrence of this group in the African continent. Moreover, a dispersal from North America to Africa—potentially via Europe—in the Jurassic would be consistent with the similarities among Late Jurassic terrestrial faunas of these continents [64,66]. Given the long evolutionary history of osteoglossiforms in Africa, it is perhaps surprising that fossils of these fishes are almost absent from South American Mesozoic deposits, as South America and Africa were joined into a single continental landmass until the beginning of the Late Cretaceous, around 100 Ma [67]. The only exception is represented by †*Laeliichthys*, a close South American relative of notopterid knifefishes, a clade that today inhabits only Africa and Southeast Asia. Crown notopterids are reconstructed as ancestrally African like other osteoglossiforms, and they probably dispersed from Africa to the Indian subcontinent in the Late Cretaceous across a narrow Mozambique Channel [25,26].

The last common ancestor of all osteoglossids (extant and extinct) included in this analysis is inferred to have been marine, probably descending from an African freshwater lineage of osteoglossiforms (with some uncertainty between African and North American origin under the alternative biogeographical scoring scheme; see electronic supplementary material). All three major clades within Osteoglossidae (†Phareodontinae, Arapaiminae and Osteoglossinae) have likely originated from marine ancestors, and reinvaded freshwater habitats multiple times independently in different continents—including North America, Asia, Australia and South America. Though starkly in contrast with traditional views of bonytongue biogeography (e.g. [7]), these results strongly support recent hypotheses of marine dispersal as the biogeographical process responsible for the disjunct distribution of extant osteoglossid bonytongues [31,37,38]. The four-to-five independent transitions towards freshwater environments from the marine realm reconstructed from BSMs (figure *3b,c*) are very likely to be an underestimate. This is due to the somewhat simplistic nature of the DEC + *j* model used for the biogeographical analysis, which assumes that the per-lineage dispersal rates between regions are all equal, symmetric, and constant through time, and does not penalize direct dispersal between very distant freshwater regions. For example, the two closely related Eocene freshwater genera †*Phareodus* and †*Phareoides* are, respectively, from the western United States and from Australia, and our analysis reconstructs their ancestral geographical area to be either North America or Oceania, implying a direct long-distance dispersal from one to the other without passing through a marine stage. Given that these genera are nested within a marine clade (figure 2), it is not unreasonable to hypothesize that they might instead represent two independent freshwater invasions from marine ancestors, not captured by the DEC + *j* model. A similar reasoning might be applied to the reconstructed ancestral areas of crown Osteoglossinae and crown Arapaiminae (see §3). Thus, we predict that biogeographical models downweighting direct dispersal between distant freshwater regions would recover an even more pre-eminent role of marine dispersal and marine-to-freshwater transitions in the biogeographical history of bonytongue fishes.

### (b) Marine origin and dispersal in bonytongue fishes

Speculation on marine dispersal in osteoglossid bonytongues began with recognition of the Eocene †*Brychaetus* as an osteoglossid [34]. Several more taxa have been subsequently described from marine deposits, all restricted to the early Palaeogene [35,36,68]. However, the lack of a phylogenetic framework and the uncertain systematics of these marine forms hindered any formal test of the marine dispersal hypothesis. Until now, the strongest arguments in favour of marine dispersal in osteoglossids came from estimated divergence times younger than the continental fragmentation of Gondwana [37,38,69], and from the observation that closely related Palaeogene taxa—sometimes even classified in the same genus—have been found in freshwater deposits as distant as Wyoming is from Australia [31]. Here we provide direct fossil evidence for marine dispersal in the lineage leading to extant osteoglossid bonytongues—arowanas and arapaimas. We find that, rather than forming a single clade or being randomly interspersed across bonytongue phylogeny, marine bonytongues form a ‘cloud’ at the base of Osteoglossidae from which all three major osteoglossid subclades (Arapaiminae, Osteoglossinae and †Phareodontinae) emerged. Ancestral state reconstructions strongly support a single freshwater-to-marine transition on the osteoglossid stem, followed by at least four—but likely more—independent marine-to-freshwater reversals. This result is remarkable for several reasons. First, to the authors’ knowledge, this is the first time that a group (crown Osteoglossidae) whose extant members and closer extant relatives are all exclusively freshwater is reconstructed as ancestrally marine. Second, major environmental transitions such as the freshwater-to-marine one are rare—albeit not unlikely—in teleost fish groups [70,71]. Third and finally, this reconstruction implies that several distinct lineages of osteoglossids were wiped out from marine environments around or soon after the middle Eocene, and that these fishes never reinvaded the sea afterwards. More palaeontological data would be needed to test whether competition with other predatory fishes such as several acanthomorph lineages that diversified around the same time [72], or severe climate change towards colder temperatures in the middle Eocene–Oligocene interval [73], played some role in the demise of marine bonytongues.

Because occurrences of bonytongue fossils in marine deposits are mostly restricted to the early Palaeogene (figure 1), it has been previously suggested that the evolution of marine bonytongues might have happened in the wake of the Cretaceous–Palaeogene (K–Pg) mass extinction that wiped out several large predators [74] and triggered the diversification of new clades, including most modern lineages of marine piscivorous fishes [29,75]. However, our total-evidence analysis recovers a much older origin of marine bonytongues, with the ancestral osteoglossid—reconstructed as marine—originating deep in the Cretaceous, at least 90 million years ago (figure 2). Several factors—not mutually exclusive—might explain this 25 Myr discrepancy between the oldest known marine bonytongue fossils and the youngest inferred age for the ancestral osteoglossid. It is possible that bonytongues invaded marine environments early in the Cretaceous but remained geographically and/or ecologically restricted for several million years, until the K–Pg mass extinction. If that was the case, then their absence from the Cretaceous marine fossil record could be more easily explained by regional (rather than global) patterns of the marine fossil record. Our results suggest that the bonytongue lineage leading to osteoglossids and to the freshwater-to-marine transition was likely African (although this is more uncertain under our alternative biogeographical scoring scheme; see electronic supplementary material), and the marine fossil fish record of the Late Cretaceous of Africa is extremely limited—virtually non-existent in Sub-Saharan Africa [76]. Another possible explanation for the age discrepancy pertains to the tree model employed for our total-evidence phylogenetic analysis, which assumes constant diversification rates through time and among lineages. If the environmental transition at the base of Osteoglossidae triggered an increase in the diversification rate of the group compared with other bonytongues, then a constant diversification model would likely overestimate the origin age of osteoglossids.

### (c) Impact of fossil data on biogeographical inference

Bonytongue fishes represent a striking case study of how the inclusion of fossil data can dramatically alter biogeographical inference for extant organisms. Such an outcome might happen for two main reasons: (i) extinct taxa might be found in geographical areas outside the modern biogeographical range of the clade of interest; (ii) extinct taxa might display eco-morphological characteristics that are outside the spectrum of adaptations found in extant representatives of the same clade. In the case of bonytongue fishes, both reasons apply: extinct bonytongues have been found in Europe and continental Asia, where they are completely absent today; and bonytongue fossils have been found in marine deposits, demonstrating a much broader ecological tolerance in the past than today. The fossil record has arguably caught osteoglossid bonytongues in the act, providing a snapshot of their marine-adapted evolutionary history which would have otherwise remained concealed. Face-value interpretation of biogeographical patterns derived exclusively from modern distributions can lead to a partial—if not outright wrong—inference by ignoring a key data source like the fossil record, as showcased by the results of our biogeographical analysis when excluding extinct taxa (figure 3*a*; electronic supplementary material, figures S3 and S4).

Despite the obvious relevance of fossil data, some limitations remain to their inclusion in phylogeny-based biogeographical studies. First, fragmentary fossils are often not included in tip-dating phylogenetic analyses because they cannot be scored for the vast majority of characters in a morphological matrix. However, fragmentary specimens still provide useful biogeographical information if they can be at least assigned to a broad taxonomic level (such as family or order). For example, they can record the first or only occurrence of a clade in a certain geographical area. This information is lost when including only more complete fossils that are diagnostic at species level into biogeographical analyses. Second, current phylogeny-based biogeographical models such as DEC do not take into account spatial and temporal biases of the fossil record [77]. The effect of these biases on biogeographical analyses that include extinct taxa as tips has been explored only in limited cases [78,79] and never for DEC-like models, but it is likely that they have an impact on reconstructed ancestral areas. As biogeographical studies including fossils as sampled tips will become more common in the future, further exploration of fossil record biases in biogeographical inference will be paramount.

Occurrence-based approaches to biogeographical inference can accommodate both fragmentary fossils and spatio-temporal biases of the fossil record [80,81], but they lack phylogenetic information. Time-stratified, spatially explicit models of fossil preservation potential could be developed in a Bayesian phylogenetic framework, similarly to how the geographical structure of biomes over time and its interaction with lineage dispersal has been recently modelled [82]. At the same time, fossil occurrence data can be jointly modelled with a phylogenetic tree through the occurrence birth–death process [83], and relevant biogeographical parameters such as dispersal rates might be estimated under this framework. While a comprehensive modelling of spatio-temporal fossilization dynamics for phylogeny-based biogeographical inference will pose several technical and computational challenges, it represents a promising research avenue to properly use an invaluable data source that cannot be substituted by neontological data, as demonstrated by the case of bonytongue fishes.

## Data Availability

Morphological and molecular character matrices, MrBayes scripts, MCMCMC log and tree files, tree files of the consensus tree and the 200 sampled trees from the posterior, BioGeoBEARS and other R package scripts and BioGeoBEARS output files are available in the Dryad repository [84]. Supplementary material is available online [85].
